# Emergency Decision-making Method of Unconventional Emergencies in Higher Education Based on Intensive Learning

**DOI:** 10.1155/2022/4317697

**Published:** 2022-09-28

**Authors:** Yongjun Zhou

**Affiliations:** Minnan Science and Technology University, Quanzhou, Fujian 362332, China

## Abstract

In order to improve the emergency decision-making and management ability of unconventional emergencies in Colleges and universities, an emergency decision-making method for unconventional emergencies in Colleges and Universities based on reinforcement learning is proposed. The function of the emergency decision-making model of unconventional emergencies in Colleges and universities is constructed, and the optimal control of the emergency decision-making process of unconventional emergencies in Colleges and universities is realized by using the queuing theory model. Based on the sub-sequence decomposition method, the reinforcement learning function is optimized. Combined with big data scheduling and the empirical mode decomposition method, the effective probability density function of emergency decision-making for unconventional emergencies in higher education is calculated, and the optimal solution vector analysis of emergency management and scheduling decision-making for unconventional emergencies in Colleges and universities is realized according to parameter estimation and quantitative optimization results. The test results show that this method has a good ability to optimize and schedule the emergency decision-making of unconventional emergencies in higher education, and has strong convergence. This method improves the emergency response-ability, and the time cost is short.

## 1. Introduction

The emergency response ability of unconventional emergencies in colleges and universities is the key to emergency response at the beginning, emergency treatment in the middle, and recovery afterward. In order to reduce the casualties and property losses of unconventional emergencies in colleges and universities, ensure the normal operation of colleges and universities, and effectively allocate emergency support resources [1], it is necessary to manage the emergency response ability of unconventional emergencies in colleges and universities. In order to effectively meet the emergency needs in the new situation and improve the scientific management of unconventional emergencies in colleges and universities, it is necessary to change the corresponding management concepts and methods, pay attention to the evaluation of existing emergency capabilities from the perspective of supply and demand, and realize quantitative management. Because non-quantitative management is highly subjective, random, and unreliable, managers often evaluate, make decisions and manage through experience, which easily leads to decision-making mistakes and hidden problems [2]. Compared with non-quantitative management, quantitative management can more effectively digitize the resources required by the emergency response capability, mine the information through mathematical tools, and obtain more objective quantitative results, which can be applied to the planning, organization, coordination, and control of the emergency response process of unconventional emergencies in higher education [3].

The structure of emergency capability of unconventional emergencies in colleges and universities refers to the composition and proportion of each element of the emergency capability of unconventional emergencies in colleges and universities [4]. The elements of emergency response capability of unconventional emergencies in colleges and universities and their proportional interaction effect determine the state of emergency response capability of unconventional emergencies in colleges and universities [5]. The influencing factors of emergency response ability of unconventional emergencies in colleges and universities mainly focus on personnel quality, resource support, cooperative response, management ability, and so on. However, different response processes for unconventional emergencies in higher education have different requirements for emergency response capabilities. It is necessary to allocate emergency resources in time according to specific scenarios and adjust the proportion of emergency response capability elements of unconventional emergencies in higher education to meet specific emergency needs. From the perspective of intensive learning and dynamic emergency management and dispatch, this article studies the demand structure and supply structure of the emergency capacity of unconventional emergencies in colleges and universities, to evaluate the matching degree between the emergency capacity building of unconventional emergencies in colleges and universities and the emergency demand, so as to put forward more targeted countermeasures to improve the emergency capacity. Yang Jin believes that because some factors of emergency management ability of unconventional emergencies in higher education are uncertain or difficult to quantify, the description of emergency management execution ability is fuzzy [6]. When evaluating the emergency management execution ability of higher education, a fuzzy comprehensive evaluation method can be used to make a general and general understanding of the situation of emergency management execution ability. For some evaluation indexes that cannot be quantitatively described, the qualitative prediction method is usually used, and it is judged by the knowledge and experience of experts. Yu Yanling believes that it is very important to conduct scientific and reasonable evaluations and build a complete evaluation index system. In order to ensure the scientificity and applicability of the established system, the selection of indexes in the evaluation index system should follow certain principles, and the determination of evaluation indexes should take into account various factors. The evaluation index system established according to the above rules can become a complete, objective, scientific, and reasonable evaluation index system, and the evaluation of the evaluation index can be more effective. There are also academic disputes about the understanding of emergency response capability. Dong Shuang believes that the emergency response ability of colleges and universities includes three aspects: early warning ability, crisis response ability, and crisis recovery ability. It is necessary to analyze the resources, skills, knowledge, and other elements of emergency response capability by investigating the capability composition and functions in different stages of crisis events. It is considered that the emergency management ability of colleges and universities in Yang Bin refers to the ability of college emergency management personnel to avoid emergencies and reduce the losses caused by emergencies by mastering certain ability elements, including resources, knowledge, and skills [7]. Liu Wei believes that the emergency management of colleges and universities should form a systematic process according to the process of unconventional emergencies in colleges and universities, the people involved, and the losses caused. In this system, from the prevention of the emergency management of unconventional emergencies in higher education, to the outbreak of emergencies, to the loss of people and property, to the rescue, recovery, reconstruction, and summary learning, the principles of the emergency management process of unconventional emergencies in higher education can be completely outlined [8].

In view of the above problems, this study proposes an emergency decision-making method for unconventional emergencies in higher education based on reinforcement learning. First, the model function of emergency decision-making for unconventional emergencies in higher education is constructed. Then, according to the results of parameter estimation and quantitative optimization, the optimal solution vector analysis of emergency management and scheduling decision of unconventional emergencies in higher education is realized. Finally, the experiment verifies the good effect of this method in improving the emergency response ability of unconventional emergencies in higher education.

## 2. Reinforcement Learning Theory

**Figure 1:**
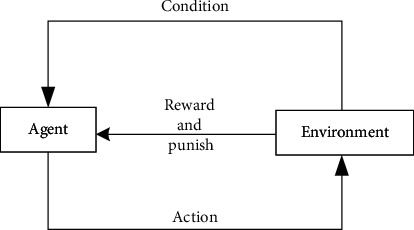
Reinforcement learning process.

Reinforcement learning, also known as reinforcement learning, evaluation learning, or reinforcement learning, is one of the paradigms and methodologies of machine learning. It is used to describe and solve the problem that agents maximize returns or achieve specific goals through learning strategies in the process of interacting with the environment. The common model of reinforcement learning is the standard Markov decision process. According to the given conditions, reinforcement learning can be divided into model-based reinforcement learning and modeless reinforcement learning, as well as active reinforcement learning and passive reinforcement learning. The variants of reinforcement learning include reverse reinforcement learning, hierarchical reinforcement learning, and reinforcement learning of some observable systems. The algorithms used to solve reinforcement learning problems can be divided into two categories: strategy search algorithm and value function algorithm. A deep learning model can be used in reinforcement learning to form deep reinforcement learning. Inspired by behavioral psychology, reinforcement learning theory focuses on online learning and tries to keep a balance between exploration and utilization. Different from supervised learning and unsupervised learning, reinforcement learning does not require any data to be given in advance but obtains learning information and updates model parameters by receiving reward feedback from the environment. If an agent's behavior strategy leads to a positive reward reinforcement signal in the environment, then the agent's tendency to produce this behavior strategy will be strengthened in the future. The goal of Agent is to find the optimal strategy in each discrete state to maximize the expected discount reward. Reinforcement regards learning as a tentative evaluation process. The agent selects an action for the environment, and the environment changes its state after accepting the action. At the same time, a reinforcement signal is generated and fed back to the agent. The agent selects the next action according to the reinforcement signal and the current state of the environment. The principle of selection is to increase the probability of positive reinforcement. The selected action not only affects the immediate enhancement value but also affects the state of the next moment in the environment and the final enhancement value. The specific operation process is shown in Figure 1.

According to the reinforcement learning process shown in Figure 1, with the advantages of high sample utilization, small value function estimation and variance, and convenient falling into local optimization, this article studies the emergency decision-making method for unconventional emergencies in higher education.

## 3. Analysis of the Quantitative Model Framework of Emergency Response Capacity of Unconventional Emergencies in Colleges and Universities

### 3.1. Applicability Analysis of Quality Function Deployment Theory

The needs of emergency management in colleges and universities, that is, in the process of emergency management, the goal of achieving the ideal level of state control of emergencies in colleges and universities can also be called demand goals, such as short response time, small accident impact range and fast information reporting. In the process of emergency management, we can quantitatively analyze the degree of demand satisfaction, which is an analysis of the emergency management process [9–11].

The supply of emergency capacity in colleges and universities needs to meet the needs and objectives of emergency management. Meeting the needs and objectives of emergency management depends on the supply of emergency capability, which is provided by the emergency management department of colleges and universities. The emergency management department of colleges and universities is usually not a fixed department, but is composed of emergency managers from the principal's office, public security department, school hospital, student affairs office, and other departments. The supply of emergency capability is usually reflected in decision-making and command, resource guarantee, rehabilitation, reconstruction, etc., which has a certain structural relationship. It is necessary to clarify the key points of emergency capability construction [12, 13].

If the supply of emergency capability can guarantee the realization of the demand target in the emergency treatment process, the emergency can be controlled according to the demand target; If the supply of emergency capacity cannot guarantee the realization of the demand target in the process of emergency treatment, it is necessary to adjust the structure of relevant emergency capacity and improve the level of emergency capacity [14].

**Figure 2:**
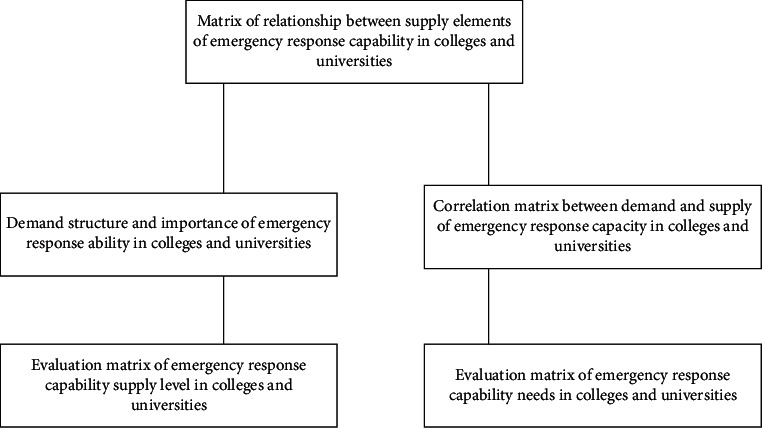
Overall framework of a quantitative model of emergency response capacity in colleges and universities.

As the emergency management and emergency response capability of colleges and universities show the relationship between demand and supply, the theory of quality function deployment and its quality manifestation can be used for analysis. The overall framework of the quantitative model of emergency response capacity in colleges and universities established in this study refers to the method of quality function development and the principle of building house of quality, combines the specific demand and supply environment of emergency response capacity in colleges and universities, and takes the matching management of supply and demand as the guiding ideology [15, 16]. On the basis of analyzing the demand structure and importance of emergency response capacity, the relationship between supply elements of emergency response capacity, and the relationship between supply and demand of emergency response capacity, it evaluates the demand achievement and supply level of emergency response capacity in colleges and universities. The overall framework of the quantitative model of college emergency response capacity proposed in this study is shown in Figure 2.

### 3.2. University Education Unconventional Emergency Decision-Making Structure Model

The function of the emergency decision-making model for unconventional emergencies in higher education is constructed, and the queuing theory model is used to optimize the process of emergency decision-making for unconventional emergencies in higher education. The database of emergency decision-making for unconventional emergencies in higher education consists of four parts: database server, information management middle ware, data storage server, and browser [17]. When constructing the emergency decision-making model of unconventional emergencies in higher education, the simulation environment is preset, and the specific parameters are:

  Virtual machine VM10.0
  Processor AMD A4-4300 M 2.5 GHz × 1
  Memory 3 GB
  Graphics AMD
  Hard disk 50 GB
  Operating system ubuntu 15.04 64 bits
  Kernel version 3.19.0-15-generic
  Search engine toolkit Lucene2.4.3.

**Figure 3:**
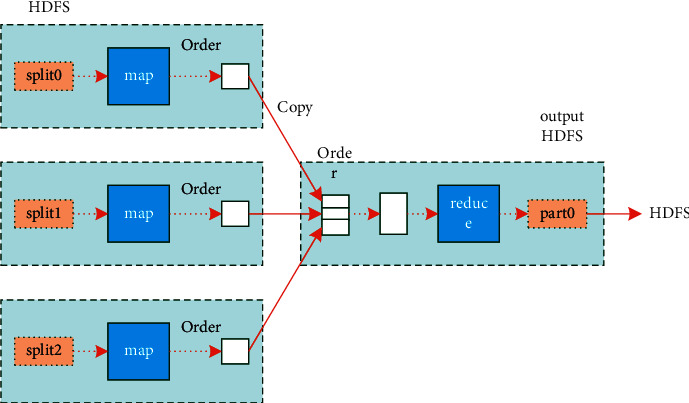
Execution process of emergency decision-making for unconventional emergencies in higher education.

Based on the above parameters, in cloud storage, the implementation process of emergency decision-making for unconventional emergencies in university education is shown in Figure 3.

In Figure 3, it is assumed that *U*_*i*_ is the autocorrelation data of the information interaction data set of the multi-feature cloud storage database for emergency decision-making of unconventional emergencies in higher education, and the related function data of each unstructured data set are independent of each other. Among them, the resource utilization rate of the whole emergency decision-making platform for unconventional emergencies in higher education is mentioned in the formula given below:

(1) $$\begin{array}{c} \lambda_{SRm}=\sum_{i=1}^{M}\lambda_{i}p_{im}, \end{array}$$

where *λ*_*i*_ is the basic file block of emergency decision-making for unconventional emergencies in higher education, and *p*_*im*_ is the decision probability. Using the model of queuing theory, the specific characteristic values of emergency decision-making scheduling for unconventional emergencies in higher education are obtained. The demand factor values of emergency capability in higher education are mentioned in the formula given below:

(2) $$\begin{array}{c} I_{SRm}=\frac{L_{SRm}}{\rho_{SRm}}=\frac{\sum_{i=1}^{M}\lambda_{i}p_{im}\left(T_{\text{wait}}+T_{\text{service}}\right)}{\rho_{SRm}}, \end{array}$$

where *L*_*SRm*_ is the emergency capability parameter of colleges and universities, *ρ*_*SRm*_ is a preliminary parameter for sorting, summarizing, analyzing and adjusting. *p*_*im*_ is the emergency dispatch factor of unconventional emergencies in colleges and universities, *T*_wait_ is the waiting time, and *T*_service_ is the service time of emergency dispatch of unconventional emergencies in colleges and universities. Assuming that the emergency decision of unconventional emergencies in colleges and universities adopts unstructured data sets for feature classification, The classified aggregated tag information conforms to the mixed distribution of distribution factors, and the auto-correlation quantum sequence of tag information of file blocks is obtained by numerical calculation, thus realizing the emergency dispatch of unconventional emergencies in higher education and the optimized access to the database. The elements obtained through various channels are preliminarily sorted out, summarized, analyzed and adjusted. In the emergency management of unconventional emergencies in higher education, the difference in meaning expression caused by the difference in conceptual categories and semantics should be avoided, and it should be adjusted to a structured language [18, 19].

### 3.3. Sub-Sequence Decomposition of Emergency Decision-Making for Unconventional Emergencies in Higher Education

On the basis of the overall design of the structural model of emergency decision-making for unconventional emergencies in higher education, the subsequence decomposition of emergency decision-making for unconventional emergencies in higher education is carried out. By analyzing the traditional methods, it can be seen that the existing emergency decision-making schemes for unconventional emergencies in higher education have the following shortcomings: (1) they can only be verified a limited number of times; (2) Most emergency decision-making schemes for unconventional emergencies in higher education are based on coding technology. In this study, the sub-sequence decomposition method of unconventional emergency decision-making in university education is used to logically encode and decrypt the cloud data of the receiving node of large-scale cloud dynamic data.

The solution principle of the subsequence decomposition method is as follows:

If the head of a continuous subsequence is negative (that is, the continuous subsequence starting from the starting point), the sum of the sequence obtained by dropping the head is larger. Therefore, whenever there is a header and a negative, set the sum to 0, which means that the cumulative sum is renewed. After updating the sum, the value of the maximum sum is also updated. The pseudocode is expressed as:

  int sum = 0;
  int res = -INF;
  for (int *i* = 0; *i* < *N*; *i*++) {
  sum + = nums [*i*];
  res = math. max (res, sum);
  if (sum <0)
  sum = 0;
  }

According to the solution process of the above sub-sequence decomposition method, the job execution information is obtained, and the new mapping formed in the multi-source nodes is obtained [20], as shown in the formula as follows:

(3) $$\begin{array}{c} x_{n}={\left[x\left(0\right),x\left(1\right),\ldots ,x\left(N-1\right)\right]}^{T}, \end{array}$$

where *x*(0), *x*(1),…, *x*(*N* − 1) represents the time series of emergency decision-making for unconventional emergencies in higher education. In the demand structure table of emergency capability in higher education, the tag information is related to *S*-Table. And *h*(·) is the broadcast data packet. Assuming that the emergency decision of unconventional emergencies in higher education has correctly received the broadcast information packets of *d*(0 ≤ *d* ≤ *n*) original data streams, if the receiving node has received the membership samples of (*g*_1_, *Y*_1_), (*g*_2_, *Y*_2_),…, neighbor samples from the broadcast source, the retransmission packet *X*_1_, *X*_2_,…, *X*_*d*_ neighbor samples of the scheduling factor of unconventional emergencies in higher education is obtained. For simplicity, set the subsequence of emergency decision-making for unconventional emergencies in higher education that *W*_*c*_ < *δ*(*t*_*c*_, *t*_*a*_) has correctly received in the current window of the emergency decision-making process for unconventional emergencies in higher education, and the subsequence is *X*_*d*+1_, that is, the lost data packet is *W*_*c*_ < *δ*(*t*_*c*_, *t*_*a*_), and the weighting coefficient, and there is a relationship of given as the formula given below:

(4) $$\begin{array}{c} \delta \left(t_{c},t_{a}\right)=\frac{2^{-\lambda \left(t_{c}-t_{a}+T_{d}\right)-1}}{2^{-\lambda T_{d}}-1}, \end{array}$$

where *t*_*c*_ is the time series of *A*[*n*+1] tuples, and *t*_*a*_ is the data decision fusion granularity of the isolated core cluster of emergency decisions for unconventional emergencies in higher education. The realization steps of sub-sequence decomposition to obtain emergency decisions for unconventional emergencies are described as follows:

(1) according to *S*_*k*_ model *M*_*k*_, calculate the predicted value of emergency decision of unconventional emergencies in higher education, search the hierarchical data structure tree composed of internal nodes of database access buffer through granularity decomposition of data information, and initialize the reinforcement learning parameters of emergency decision of unconventional emergencies in higher education;
(2) until the specified iteration number *k* is reached or the termination condition is met, the value of is updated to $\overset{⌢}{\theta }$, and the sub-sequence decomposition of the emergency decision of unconventional emergencies in higher education is carried out, and the decision criteria of whether the decomposition is accepted or not are obtained as shown in formula below:
  (5) $$\begin{array}{c} \underset{a,\tau }{\max}\left|\int_{0}^{T}r\left(t\right)\frac{1}{\sqrt{a}}f^{*}\left(\frac{t-\tau }{a}\right)\text{d}t\right|=\underset{a,\tau }{\max}\left|W_{f}r\left(a,\tau \right)\right|H_{1}>\lambda \lambda <H_{0}. \end{array}$$
(3) When the data stream of emergency decision-making for unconventional emergencies in higher education is processed, the node *DB*_*ij*_ is locked and inserted into the node *f* through the < key, pointer > instruction, so as to realize the scheduling of emergency decision-making for unconventional emergencies in higher education. The execution time of emergency decision-making for unconventional emergencies in higher education is set to (*t*_current_+*ϕ*_*k*_/*d*_*x*_/*d*_*t*_).

**Figure 4:**
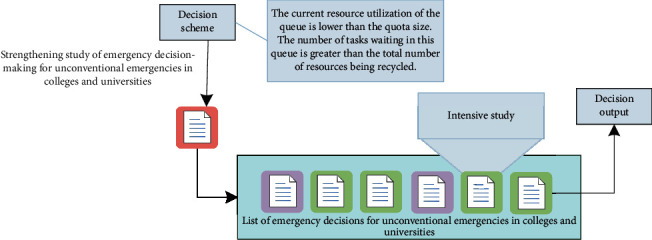
Sub-sequence decomposition of emergency decision-making for unconventional emergencies in higher education.

To sum up, the subsequence decomposition process of emergency decision-making for unconventional emergencies in higher education in cloud storage systems is shown in Figure 4.

## 4. Improved Implementation of Algorithm

The intensive tracking learning and adaptive optimization control in the emergency decision-making process of unconventional emergencies in colleges and universities have been established, the effective probability density function of emergency decision-making for unconventional emergencies in higher education is calculated, and the optimal solution vector analysis of emergency management and scheduling decision-making for unconventional emergencies in higher education according to parameter estimation and quantitative optimization results is realized, which leads to the response of emergency decision-making for unconventional emergencies in higher education.

In order to overcome the disadvantages of traditional methods, this study proposes an emergency decision algorithm for unconventional emergencies in higher education based on subsequence fuzzy decision-making. Fuzzy decision is a decision with fuzzy elements (such as criteria and alternatives). Fuzzy decision-making method refers to the use of fuzzy mathematical methods to deal with some complex decision-making problems. This kind of problem generally has the characteristics of a large-scale system. The relationship between systems is very complex, and some variables cannot be accurately assigned. These variables belong to fuzzy factors and involve certain subjective factors, which makes the relationship between subsystems and variables unclear, so it must be handled with the help of sorting, fuzzy evaluation, and other methods. The implementation of this method mainly includes two aspects: function establishment and pattern recognition.

### 4.1. Create Function

The improved algorithm design is described as follows: In the construction of data temporal distribution, two points of database temporal attributes of emergency decision-making for unconventional emergencies in higher education are assumed to be *n*_1_, *n*_2_, and the distance between two points SD is defined as normal distribution, and the consistency index of emergency decision-making for unconventional emergencies in higher education is defined as *N*=Δ*x*^2^. In the interval [−Δ*x*/2, Δ*x*/2], sub-sequence fuzzy decisions have complete consistency. Choose different file block sizes, and get the response time of emergency decision-making homework for unconventional emergencies in higher education as shown in the formula given below:

(6) $$\begin{array}{c} Y^{*}=GXN_{0}+\frac{\tilde{x}\left(t\right)}{\tilde{x}\left(u\right)}, \end{array}$$

where $\tilde{x}\left(t\right)$ is the coding vector of emergency decision-making for unconventional emergencies in higher education, *N*_0_ is the sub-sequence fuzzy decision-making scale parameter of emergency decision-making for unconventional emergencies in higher education, and $\tilde{x}\left(u\right)$ is the reinforcement learning function. By using subspace fusion, the algorithm of emergency decision-making for unconventional emergencies in higher education is improved, and the attribute weight distribution of emergency decision-making for unconventional emergencies in higher education is as shown in the formula given below:

(7) $$\begin{array}{c} F=\left[\begin{array}{ccccc} \omega^{0} & \omega^{0} & \omega^{0} & \cdots & \omega^{0}\\ \omega^{0} & \omega & \omega^{2} & \cdots & \omega^{k-1}\\ \omega^{0} & \omega^{2} & \omega^{4} & \cdots & \omega^{2\left(k-1\right)}\\ \vdots & \vdots & \vdots & \vdots & \vdots \\ \omega^{0} & \omega^{k-1} & \omega^{2\left(k-1\right)} & \cdots & \omega^{{\left(k-1\right)}^{2}} \end{array}\right]=\prod_{0\leq j<i\leq k-1}\left(\omega^{i}-\omega^{j}\right), \end{array}$$

where *ω*^*i*^, *ω*^*j*^ are the weighted subsequences of emergency decision-making for unconventional emergencies in higher education. Assuming that the phase spectra of two sections of multi-queue management task data *x*_1_(*t*) and *x*_2_(*t*) are independent and autocorrelation, the correlation is analyzed by using the analysis method of the decision-making laboratory. By calculating the overall impact matrix of emergency capability supply, the reachable matrix is obtained, and the relationship between emergency capability supply elements is analyzed. The differential equation is expressed as shown in the formula given below:

(8) $$\begin{array}{c} \left\{\begin{array}{c} {\dot{m}}_{i}\left(t\right)=-a_{i}m_{i}\left(t\right)+b_{i}\left(p_{i}\left(t-\sigma \right),p_{2}\left(t-\sigma \right),\ldots ,p_{n}\left(t-\sigma \right)\right),\\ {\dot{p}}_{i}\left(t\right)=-c_{i}p_{i}\left(t\right)+d_{i}m_{i}\left(t-\tau \right), \end{array}\right. \end{array}$$

where *a*_*i*_, *b*_*i*_, *c*_*i*_, and *d*_*i*_ are the emergency capability supply element, *t* is the decision time, *τ* is the delay, and *m*_*i*_(*t*) is the emergency capability supply parameter.

Formula (8) is the established fuzzy decision function.

### 4.2. Pattern Recognition

In the multi-source hierarchical data structure, intensive learning of emergency decision of unconventional emergencies in higher education is carried out, and the data classification attribute is *A*={*A*_1_, *A*_2_,…, *A*_*m*_}. The information fusion center in the emergency management database of unconventional emergencies in higher education forms a relative index result $\tilde{Q}$ and a slack query result *X*_1_ ⊕ *X*_5_. In the application of XNCWBR, it is necessary to broadcast and retransmit the emergency management factor (*E*_*i*_, *E*_*j*_, *d*, *t*) of unconventional emergencies in higher education. The trust relationship of query structure is expressed as follows. According to the existing emergency management data packets *X*_1_, *X*_2_, *X*_3_, *X*_8_, *X*_7_, *X*_9_, *X*_10_ of unconventional emergencies in higher education and the received sub-sequence task modules of big data, the emergency decision of unconventional emergencies in higher education is made, and the distribution matrix of reinforcement learning state of emergency decision of unconventional emergencies in higher education is obtained (see Table 1).

### 4.3. Realize the Emergency Decision-Making of Unconventional Emergencies in Higher Education

Based on the algorithm improvement process of the above two parts, reinforcement learning algorithm is used to strengthen the emergency decision-making of unconventional emergencies in higher education, so as to realize the data scheduling in the cloud storage system. To sum up, the point cloud data analysis method, combined with big data scheduling and empirical mode decomposition, is used to establish intensive tracking learning and adaptive optimization control in the process of emergency decision-making of unconventional emergencies in higher education, and the effective probability density function of emergency decision-making of unconventional emergencies in higher education is calculated. According to the parameter estimation and quantitative optimization results, the optimal solution vector analysis of emergency management and scheduling decision-making of unconventional emergencies in higher education is realized.

## 5. Simulation Experiment

According to the idea of matching supply and demand, the supply of emergency capacity for unconventional emergencies in higher education is the core content of quantitative management of emergencies in higher education, and the basis for meeting the demand of emergency capacity for unconventional emergencies in higher education. In the experiment, through data analysis and simulation, we will study the composition of the supply of emergency capacity for unconventional emergencies in colleges and universities and the relationship between them. In the description of the satisfaction degree of emergency capacity supply elements to the demand of emergency capacity, rr can be used to indicate the degree of influence of the j-th emergency capacity supply element on the ith emergency capacity demand element, that is, the degree of correlation, which usually takes the values of 0, 1, 3 and 9, respectively, indicating strong correlation, medium correlation, weak correlation, and irrelevance. See Table 2 for the distribution of the weight factors of the importance of emergency demand objectives in colleges and universities.

According to the parameters and demand capacity allocation in Table 3, strengthen tracking learning and adaptive optimization control in the process of emergency decision-making for unconventional emergencies in higher education are established, and the effective probability density of emergency decision-making for unconventional emergencies in higher education is calculated. The calculation results of demand importance, target value, and improvement ratio are shown in Table 3.

**Figure 5:**
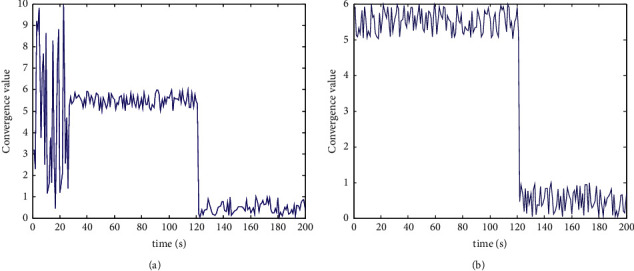
Convergence curve of emergency decision (a) before improvement (b) after improvement.

**Figure 6:**
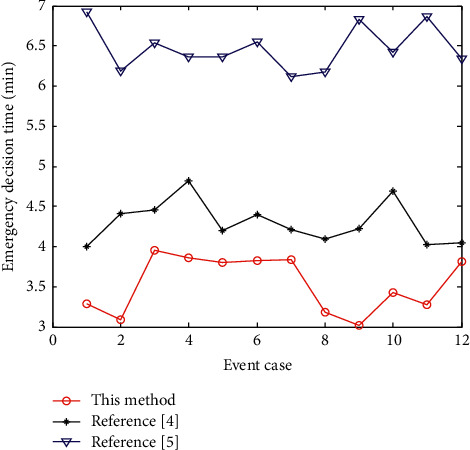
Time expenditure of emergency response to unconventional emergencies in higher education.

The importance of the supply elements of the emergency response capability for unconventional emergencies in colleges and universities needs to be transformed by “demand-supply,” so as to find out the key emergency response capability. Convert the degree of demand achievement in the supply level of emergency capacity, get the promotion ratio, build and develop the emergency capacity level pertinently, and get the convergence curve of emergency decision as shown in Figure 5, and the cost of decision time as shown in Figure 6.

Figure 5 shows that before the improvement, the emergency decision fluctuated greatly and the convergence was poor. The oscillation range was mainly divided into three parts: 0∼10, 5∼6, and 0∼1. Using this method to deal with emergencies in Colleges and universities can quickly converge to the minimum oscillation range, which is mainly divided into two parts: 5∼6 and 0∼1. Therefore, the proposed method has good convergence.

Figure 6 shows that using this method to make emergency decisions for unconventional emergencies in colleges and universities has better optimization scheduling ability, stronger convergence, improved emergency response ability of unconventional emergencies in colleges and universities, and shorter time cost. The data packet loss rate is lower than 4, which is lower than 5 and 7 in reference [4] and reference [5], which verifies that the proposed method has a better decision-making effect.

In order to further test the decision-making performance of the proposed method, this method is run in a university from June 1 to June 12, 2021 to generate Unconventional Emergency Decision-making schemes in this time period. The generation of the decision-making scheme of the system on the 12th day of operation is shown in Table 4.

Table 4 test results show that this method can be used to generate unconventional emergency decision-making schemes with a low failure rate. When the failure rate is less than 0.003% and the number of concurrent users is 385, the response time generated by the unconventional emergency decision scheme is less than 500 ms. According to the test results in Table 4, this method has ideal operation performance.

## 6. Discuss

The process management of emergency response in colleges and universities is helpful to find out the weak links in the management process. Instead of just judging by the results, it is necessary to objectively evaluate the situation of each stage in the process of emergency response. Analyze and comprehensively evaluate the disposal process of emergency management. For example, the research of this study is to achieve the goal based on the demand in the process of emergency handling, and then comprehensively evaluate the quantitative score of handling and the emergency capability supply structure that matches it after the structural transformation. To establish the concept of emergency process management, colleges and universities need to pay attention to the record of the emergency management process, form the system of database construction, and experience the exchange of emergency treatment process, so as to carry out emergency personnel training more pertinently and continuously improve the management level of emergency treatment process.

In the information age, the implementation of quantitative management is an important symbol of the contemporary management level. The essence of quantitative management is the refined utilization of resources, and it is an important concept for the management effectiveness of enterprises, units, and organizations. In essence, the process of quantitative management of emergencies in colleges and universities is based on the quantitative evaluation of the objectives of emergency needs, and the structure of the supply elements of emergency capabilities, their importance, the upgrading objectives, and supply status of emergency capabilities are obtained, so as to improve and upgrade the important emergency capabilities to better meet the needs. In addition, the quantification of the emergency response ability of colleges and universities is conducive to the realization of digitalization and indexing with the help of computer programming, and it is convenient to fully tap and utilize valuable information in the data. To strengthen the concept of quantitative management in colleges and universities, we should carry out key activities such as safety culture education, case quantitative analysis, personnel training, assessment, etc., so that the quantitative thinking mode can go deep into all levels and all kinds of emergency personnel and form a benign normative thinking.

## 7. Conclusions

In view of the long response time of traditional emergency decision-making methods, this study proposes an emergency decision-making method for unconventional emergencies in higher education based on reinforcement learning. Based on the big data distribution structure model, node resources are allocated through multi-queue decision fusion. The sub-sequence decomposition method is used to logically encode the receiving nodes of large-scale cloud dynamic data, decrypt the data, and obtain job execution information. Fuzzy decision-making method is used to improve the emergency decision-making algorithm for unconventional emergencies in higher education. The simulation results show that the algorithm has good decision fusion performance of big data subsequence. This method improves the response-ability of state characteristics in the process of data resource scheduling and mining and reduces the packet loss rate in the process of big data transmission and storage.

## Figures and Tables

**Table 1:** Emergency decision-making of unconventional emergencies in higher education strengthens learning state response.

	*X*_1_	*X*_2_	*X*_3_	*X*_4_	*X*_5_	*X*_6_	*X*_7_	*X*_8_	*X*_9_	*X*_10_
*R*_1_	*Y*	*X*	*X*	*Y*	*Y*	*X*	*X*	*Y*	*X*	*Y*
*R*_2_	*X*	*Y*	*X*	*Y*	*Y*	*X*	*X*	*X*	*Y*	*Y*
*R*_3_	*X*	*X*	*Y*	*Y*	*Y*	*X*	*X*	*X*	*X*	*X*
*R*_4_	*Y*	*X*	*Y*	*Y*	*X*	*Y*	*X*	*Y*	*Y*	*Y*
*R*_5_	*X*	*Y*	*X*	*Y*	*Y*	*X*	*X*	*X*	*X*	*Y*

**Table 2:** Distribution of weight factors of the importance of emergency demand objectives in colleges and universities.

Bound variable	Weight factor
Counselors found the accident at the first time.	0.69
Personnel are sent to hospital for rescue in time.	0.93
Protection of the scene is complete.	0.75
Accident information is reported in time.	0.06
Relevant information of the deceased is collected comprehensively.	0.23
Prevent information diffusion	0.27
Accurate event characterization	0.33
Emergency plan starts quickly.	0.10
Effective leadership decision-making	0.17
Reasonable allocation of emergency resources	0.85
Family reception in place	0.57
Network public opinion guidance	0.24
Prevent mass incidents	0.24
Prevent derivative events	0.98
Low social influence	0.24
Authoritative information release	0.08
Information transmission is smooth.	0.79
End the accident as soon as possible.	0.84
Reasonable compensation or assistance compensation to family members	0.96
Restore normal teaching order on campus on time.	0.06
Timely psychological intervention for students.	0.79

**Table 3:** Calculation results of emergency decision parameters for unconventional emergencies in higher education.

Ability requirement	Importance of demand	Target value	Improvement ratio/%
Counselors found the accident at the first time.	0.38	0.64	91.47
Personnel are sent to hospital for rescue in time.	0.22	0.90	18.31
Protection of the scene is complete.	0.56	0.96	65.72
Accident information is reported in time.	0.64	0.16	69.70
Relevant information of the deceased is collected comprehensively.	0.57	0.86	92.48
Prevent information diffusion	0.35	0.00	24.26
Accurate event characterization	0.27	0.87	3.19
Emergency plan starts quickly.	0.40	0.96	68.58
Effective leadership decision-making	0.35	0.86	10.43
Reasonable allocation of emergency resources	0.62	0.11	18.22
Family reception in place	0.05	0.29	8.35
Network public opinion guidance	0.91	0.14	12.90
Prevent mass incidents	0.46	0.33	68.15
Prevent derivative events	0.60	0.58	10.16
Low social influence	0.22	0.59	11.16
Authoritative information release	0.43	0.39	0.69
Information transmission is smooth.	0.54	0.98	56.26
End the accident as soon as possible.	0.72	0.96	7.90
Reasonable compensation or assistance compensation to family members	0.89	0.74	89.98
Restore normal teaching order on campus on time.	0.92	0.98	99.90
Timely psychological intervention for students.	0.17	0.90	29.69

**Table 4:** Generation of decision schemes.

Date	Number of decision schemes generated/piece	Successfully generated quantity/piece	Failed generation quantity/piece	Maximum concurrent users/	Maximum response time/ms
June 1st	125814	125811	3	385	436
June 2nd	214581	214579	2	289	341
June 3rd	185161	185159	2	348	285
June 4th	194513	194512	1	297	364
June 5th	234568	234565	3	264	294
June 6th	187415	187413	2	285	345
June 7th	195462	195461	1	316	294
June 8th	224687	224685	2	352	258
June 9th	269784	269783	1	348	378
June 10th	216485	216483	2	296	465
June 11th	198541	198538	3	348	418
June 12th	132548	132547	1	297	395

## Data Availability

The raw data supporting the conclusions of this article will be made available by the authors, without undue reservation.
